# Postoperative radiotherapy-induced leiomyosarcoma in breast cancer: a case report and literature review

**DOI:** 10.1007/s12282-020-01050-x

**Published:** 2020-01-11

**Authors:** Yong Liu, Jiaming Wang, Rong Su, Yi Tang, Xiaoli Liao

**Affiliations:** 1 Department of Breast Surgery, The Third Affiliated Hospital of Guangzhou Medical University, Guangzhou, 510150 Guangdong China; 2 Department of Radiotherapy, The Third Affiliated Hospital of Guangzhou Medical University, Guangzhou, 510150 Guangdong China

**Keywords:** Breast cancer, Radiotherapy, Leiomyosarcoma

## Abstract

Radiation-induced sarcoma (RIS) is a late complication of breast cancer radiotherapy, with a very low incidence, long latent period and poor prognosis. Among them, leiomyosarcoma after radiation is an extremely rare radiation-associated sarcoma. In this study, we report a case of radiation-induced leiomyosarcoma 2 years after postoperative radiotherapy for breast cancer. After the diagnosis of leiomyosarcoma, the patient received radical resection of the left chest wall leiomyosarcoma. The patient showed no relapse or progression during the follow-up time of 1 year after operation. Rare occurrence of RIS induced by postoperative radiotherapy in breast cancer and limited experience concerning its diagnosis and treatment bring obstacle to both patients and doctors. Preoperative examinations must be carefully refined. With a perfect coordination between different disciplines, RIS induced by postoperative radiotherapy in breast cancer can be ideally treated with better prognosis.

## Introduction

Radiation-induced sarcoma (RIS), also known as radiation-associated sarcoma (RAS), is a kind of soft tissue sarcoma that originates from the radiation area. It is a relatively rare complication with an incidence of 0.03–8% [1–5]. In 1922, Beck [6] first reported the case of osteosarcoma after the breast cancer radiotherapy. The histological type of most RIS is angiosarcoma [7, 8]. There is a long latent period between the occurrence of RIS and radiation therapy, usually 10 years after radiotherapy [9]. Due to this reason, RIS is prone to missed diagnosis and misdiagnosis. Because of the rarity of RIS, there is limited experience concerning its diagnosis and treatment. Establishing an accurate diagnosis is very important in planning treatment. To the best of our knowledge, we present a rare case involving radiation-induced leiomyosarcoma following a postoperative radiotherapy for breast cancer. In this study, the patient underwent radical and extensive local resection, and no obvious tumor recurrence and metastasis have been found so far.

## Case report

**Fig. 1:**
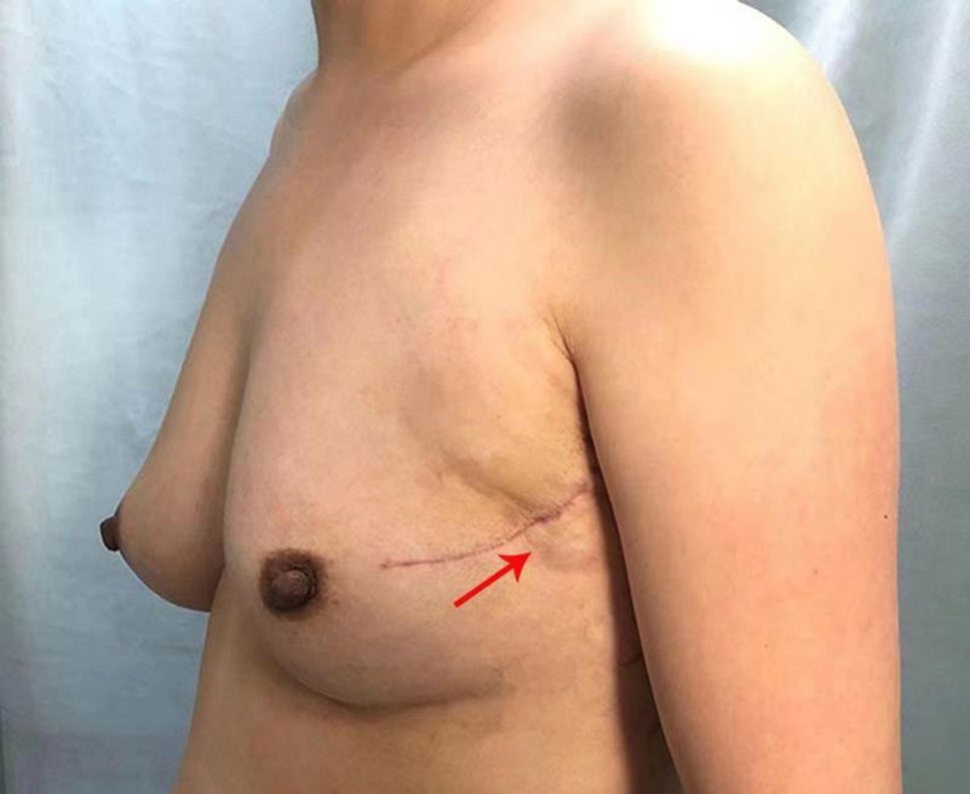
An oval mass that was 2.0 × 1.5 cm in size on the chest wall of the original radiotherapy field below the left axilla (shown by arrow)

**Fig. 2:**
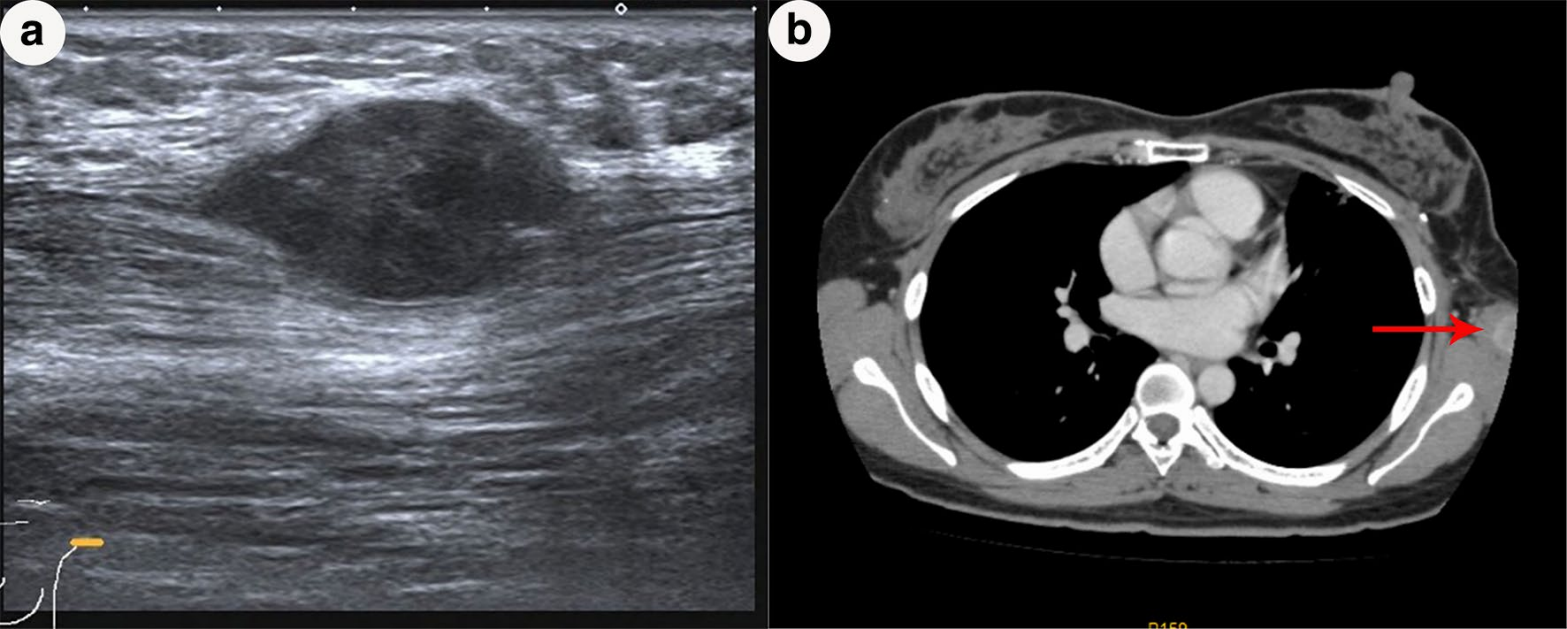
The mammary gland ultrasound showed a solid mass of the chest wall below the left axilla (**a**); chest CT showed a low-density shadow in the anterior part of the left latissimus dorsi, and the enhanced scan was significantly enhanced (arrow, **b**)

**Fig. 3:**
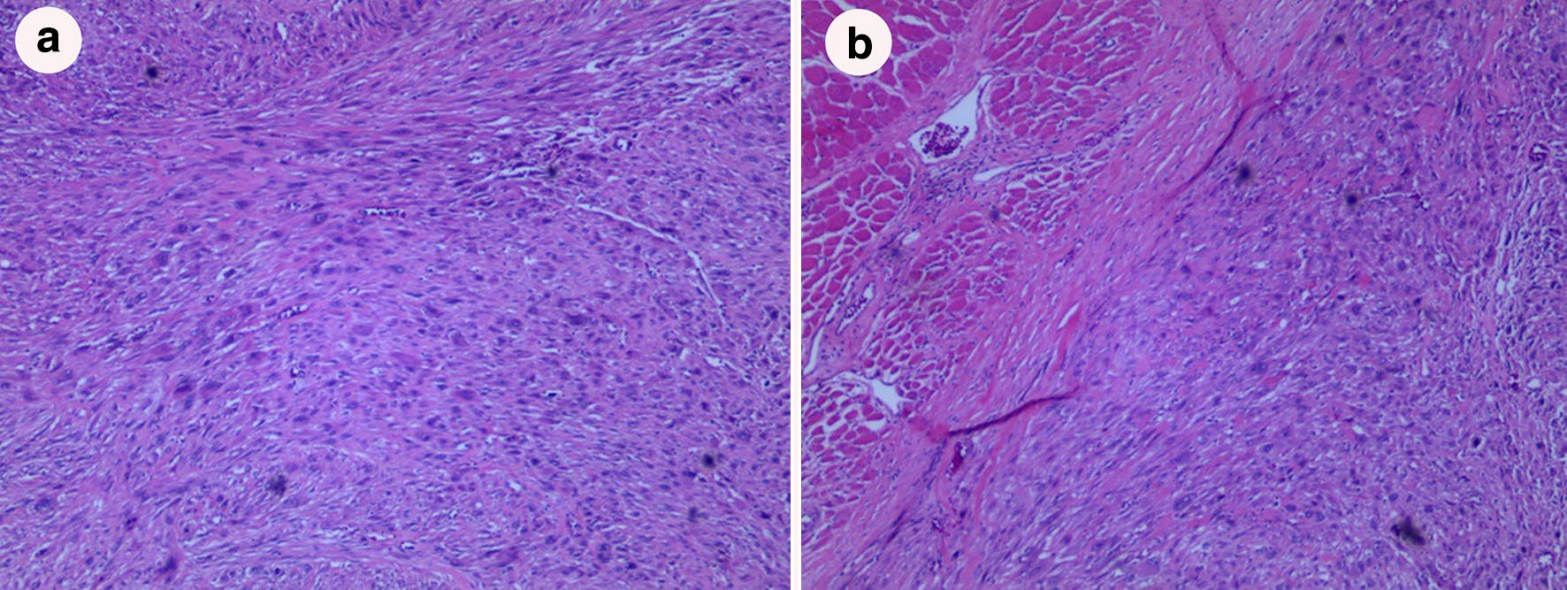
Pathological microscopy showed that the spindle cells were arranged in a strip or braid, showing giant cells and mitosis (HE, × 40, **a**); tumor cells infiltrating in the striated muscle tissue (HE, × 40, **b**)

A 28-year-old woman was referred to our hospital in July 2015 due to the discovery of an irregular mass in the upper outer quadrant of the left breast. The size of the mass was about 2 cm × 1 cm. Vacuum-assisted rotation-assisted biopsy of the left breast mass was performed. Postoperative pathology confirmed the infiltrating carcinoma of the left breast. The patient underwent breast-conserving surgery and left axillary sentinel lymph node biopsy. Postoperative pathological shows left breast invasive carcinoma, non-specific, histologic grade II, scores 7 (glandular component scores 3, nuclear atypia scores 2, fission scores 2) (modified black nuclear classification method). Infiltration focal is about 0.5 cm in diameter. The surrounding breast tissue is accompanied by more acne ductal carcinoma in situ, and involving the lobule. Left axillary sentinel lymph node showed no cancer metastasis (0/8). Immunohistochemistry: ER (70%), PR (+ 70%), Her-2 immunohistochemistry (++), FISH: Her-2 gene amplification, ki-67 (70%). One month after surgery, 8 times of adjuvant chemotherapy were completed with EC sequential TH regimen every 3 weeks. Targeted trastuzumab therapy followed for 1 year. Radiotherapy was launched immediately after the completion of chemotherapy. The dosage was simultaneously increased and strengthened. The prescription dose: CTV1 (breast): 50 Gy/1.78 gy/28 f; CTV2 (tumor bed): 60 Gy/2.14 gy/28 f. Radiotherapy range: upper border to the subclavian and second rib junction, lower border to the lower edge of the breast, no breast tissue, medial border to the sternal stem edge, no more than the midline of the sternum, lateral border to the left midline, including Latissimus dorsi. Then, regular follow-up was performed. On April 9, 2018, the patient was re-admitted to the hospital. She noticed a lump in her original radiotherapy field of the left chest wall 3 months earlier. A physical examination revealed an oval mass that was 2.0 cm × 1.5 cm in size on the chest wall of the original radiotherapy field below the left axilla. The mass showed unclear boundary, hard texture and no tenderness (Fig. 1). No masses were detected in bilateral breasts and no enlarged lymph nodes were detected in bilateral axilla. B-ultrasonography of the breast showed solid masses in the chest wall below the left axilla (Fig. 2a). After the puncture biopsy of the chest wall mass, pathology revealed a large number of spindle cells. Chest CT revealed a slightly lower density shadow in the anterior portion of the left latissimus dorsi muscle, with a CT value of about 50 HU. Progressive enhancement was evident in the enhanced scan, with a range of about 1.6 × 0.9 cm and an unclear boundary (Fig. 2b). Combined with auxiliary examination, we excluded the possibility of metastasis of other organs. The preoperative examination was completed after detailed discussion by the multidisciplinary medical team. On April 11, 2018, a local radical enlarged resection of the left chest wall leiomyosarcoma was performed. In the macroscopic pathological examination of the resected specimen: (1) a tumor that was 2 cm in diameter in size was observed. Microscopically, the tumor was composed of spindle cells which were arranged in a cord-like or braided shape with obvious pleomorphism. Giant tumor cells were easy to be seen. Pathological mitograms were visible (10/10HPF) (Fig. 3a); with coagulative necrosis; infiltrating striated muscle tissue (Fig. 3b). Immunohistochemistry: Vimentin (3+); Caldesmon (3+); SMA (3+); Ki-67 (30%); P53 (nonsense mutation); p63 (–); CK (–); MyoD1 (–); Myogenin (–). Molecular pathology: EBER (–). (2) No sarcoma was found at the edge of the tumor. According to the medical history and histopathological analysis, the tumor was diagnosed as leiomyosarcoma. Then, the patient underwent genetic testing, and the results showed no tendency for mutations in genes such as BRCA1, BRCA2, and TP53. Postoperative follow-up showed no obvious tendency of tumor recurrence.

## Discussion

Radiation therapy significantly reduces the risk of local recurrence of breast cancer and is one of the important methods for comprehensive treatment of breast cancer. Radiation-induced sarcoma (RIS), also known as radiation-associated sarcoma (RAS), is a rare soft tissue sarcoma occurring in the postoperative radiation area of breast cancer, with an incidence of 0.03–8% [1–5]. The most common histological type of radiotherapy-associated sarcoma in the literature report is angiosarcoma. Although our case does not belong to this type. Yap et al. reported that in sarcomas occurring in the radiation field, angiosarcoma accounted for 56.8%, malignant fibrous histiocytoma accounted for 15.9%, fibrosarcoma and chondrosarcoma accounted for 6.8%, and leiomyosarcoma accounted for 4.5% [7]. Sal et al. reported that among 96 patients with radiation-related sarcoma, the most common angiosarcoma accounted for 52% [8].

The incubation period between radiation-induced sarcoma and radiotherapy is 6 months to 20 years, usually 10 years after radiotherapy [10]. Therefore, many patients have delayed diagnosis due to insufficient follow-up time. The main cause of RIS is radiotherapy, its pathogenesis is thought to be due to non-fatal chromosome damage in non-tumor connective tissue cells, leading to sarcoma in the radiation field [11, 12]. Most series report radiation doses ranging from 16–112 Gy [8, 13–15], and median doses for breast irradiation are approximately 50 Gy [8, 11–15]. It has been reported that radiotherapy dose is significantly associated with the risk of subsequent sarcoma, and higher radiation doses increase the risk of bone and soft tissue sarcoma. The relative risk of radiation doses above 44 Gy is 30.6 compared to doses below 15 Gy [16].The incidence of radiosarcoma is also affected by a variety of other factors, such as BRCA1 gene mutations [17, 18], hereditary diseases (such as Gardner syndrome and Li–Fraumeni syndrome), chemotherapy with alkylating agents [19]. The most common symptoms at onset are masses, pain and changes in skin color. In this report, the onset time of the patient was 2 years after radiotherapy, and the radiation dose was 50 Gy for the breast and 60 Gy for the tumor bed, which was consistent with previous series of reports.

The current RIS diagnostic criteria (based on CAHAN's revised criteria) are: (1) the history of any benign or malignant disease; (2) years of asymptomatic incubation period; (3) Sarcoma in the past irradiation field; (4) pathology confirmed that the sarcoma is histologically different from the primary tumor [4]. Combined with the clinical features and pathological examination of this patient, the author believes that it is consistent with the diagnosis of radiation-induced sarcoma.

Radiation-induced sarcoma reported in most literature has a poor prognosis and a high recurrence rate. The average time to relapse was 12.8 months ± 10.6 (median 7.5 months) [10], the 5-year survival rate was 27–48%, the 5-year disease-free survival rate was 35%, and the average survival time was 23 months [7, 20]. The follow-up data of 12 patients with leiomyosarcoma in the Koea's study found that the diagnosis of leiomyosarcoma is a good independent predictor of multivariate analysis, which means that leiomyosarcoma is the only histological subtype with predictive value for survival. In most cases, post-radiation leiomyosarcoma is different from other breast or thoracic wall sarcomas, and it is less invasive and has a better prognosis. It is a slow-growing tumor [7]. In general, factors with poor prognosis include age, positive margin of surgery, large and highly differentiated tumors, and distant metastasis. Therefore, early detection, early diagnosis is particularly important for the improvement of RIS prognosis.

Currently, the preferred treatment for RIS is surgical resection. That is, complete removal of sarcoma and enlargement of the surrounding 2–3 cm tissue to achieve a negative margin [9, 21]. De et al. reported 46 patients with radiation-induced sarcoma who underwent different surgical resections and found that patients who underwent surgery had a significantly higher 5-year survival rate than non-surgical patients: 37% vs. 0% (*p* = 0.002), 2-year survival after radical surgery (no tumor cells under a microscope R0) was significantly higher than non-radical surgery (tumor cells were found under R1 microscope or R2 by naked eye), which is 56% and 14%, respectively (*p* = 0.02) [17].To sum up, active radical surgical resection is the most appropriate treatment after the diagnosis of sarcoma from radioactive sources.

As for adjuvant therapy, chemotherapy lacks sufficient strong evidence to support its effectiveness [22]. Van et al. included 2185 cases of soft tissue sarcomas in a large study, which were treated with chemotherapy centered on anthracyclines, achieving a 26% response rate and an average overall survival time of 51 weeks [23]. Due to the lack of relevant studies, chemotherapy with anthracyclines remains controversial. In terms of radiation therapy, because there is currently no clear evidence for the effectiveness of radiotherapy, and secondary radiotherapy will bring a lot of Toxic side effects, so for RIS patients, whether to choose radiotherapy requires clinicians to weigh the pros and cons, and make a choice after comprehensive consideration, which is not the first choice of treatment. In view of the fact that the patient had undergone local radical expanded resection, postoperative pathology showed no tumor at the resection margin, and the patient's previous history of radiotherapy, the patient was not given postoperative chemotherapy and radiotherapy after multidisciplinary discussion. After 1 year of follow-up, there was no obvious tendency for tumor recurrence.

## Conclusion

As the survival period of patients with breast cancer is greatly prolonged after comprehensive treatment, the incidence of secondary malignant tumor increases accordingly, and radiation-induced sarcoma may become more common. In conclusion, radiation-induced sarcoma (RIS) after breast cancer surgery is a late complication of postoperative radiotherapy for breast cancer, with a very low incidence, a long latent period and a poor prognosis. Due to limited case data, treatment recommendations are difficult to establish. At present, surgical resection is the mainstream treatment for radiation-induced sarcoma, and the role of adjuvant chemotherapy is still unclear. However, whether to choose radiotherapy requires clinicians to weigh the advantages and disadvantages and make a choice after comprehensive consideration, which is not the first choice of treatment. At the same time, it is emphasized that RIS requires early diagnosis, multidisciplinary treatment and close follow-up for a long time. More prospective and randomized studies are needed to provide patients with better and more standardized prognosis improvement solutions.

## Abbreviations

RIS: Radiation-induced sarcoma
RAS: Radiation-related sarcoma
EC follow TH: Epirubicin + cyclophosphamide follow docetaxel + trastuzumab
CTV1: Clinical target 1
CTV2: Clinical target area 2
Chest CT: Chest computed tomography
